# Real-world safety and efficacy of paritaprevir/ritonavir/ombitasvir plus dasabuvir ± ribavirin in patients with hepatitis C virus genotype 1 and advanced hepatic fibrosis or compensated cirrhosis: a multicenter pooled analysis

**DOI:** 10.1038/s41598-019-43554-3

**Published:** 2019-05-08

**Authors:** Chun-Hsien Chen, Chien-Hung Chen, Chih-Lang Lin, Chun-Yen Lin, Tsung-Hui Hu, Shui-Yi Tung, Sen-Yung Hsieh, Sheng-Nan Lu, Rong-Nan Chien, Chao-Hung Hung, I-Shyan Sheen

**Affiliations:** 1 Division of Hepatogastroenterology, Department of Internal Medicine, Chiayi Chang Gung Memorial Hospital, Chiayi, Taiwan; 2 Division of Hepatogastroenterology, Department of Internal Medicine, Kaohsiung Chang Gung Memorial Hospital and Chang Gung University College of Medicine, Kaohsiung, Taiwan; 3 Liver Research Unit, Chang Gung Memorial Hospital, Keelung, Taiwan; 4 Department of Hepatogastroenterology, Chang-Gung Memorial Hospital, Linkou Medical Center, Taoyuan, Taiwan

**Keywords:** Hepatitis C, Hepatitis C, Hepatitis C, Hepatitis C

## Abstract

Paritaprevir/ritonavir, ombitasvir, and dasabuvir (PrOD) with or without ribavirin shows favorable results in hepatitis C virus genotype 1 (HCV-1) patients in terms of safety and efficacy, but real-world data remain limited for those with advanced hepatic fibrosis (fibrosis 3, F3) or compensated cirrhosis (F4). A total of 941 patients treated in four hospitals (the Keelung, the Linkuo, the Chiayi and the Kaohsiung Chang Gung Memorial Hospital) through a nationwide government-funded program in Taiwan were enrolled. Patients with HCV and advanced hepatic fibrosis or compensated cirrhosis received 12 weeks of PrOD in HCV-1b and 12 or 24 weeks of PrOD plus ribavirin therapy in HCV-1a without or with cirrhosis. Advanced hepatic fibrosis or compensated cirrhosis was confirmed by either ultrasonography, fibrosis index based on 4 factors (FIB-4) test, or transient elastography/acoustic radiation force impulse (ARFI). The safety and efficacy (sustained virologic response 12 weeks off therapy, SVR_12_) were evaluated. An SVR_12_ was achieved in 887 of 898 (98.8%) patients based on the per-protocol analysis (subjects receiving ≥1 dose of any study medication and HCV RNA data available at post-treatment week 12). Child-Pugh A6 (odds ratio: 0.168; 95% confidence interval (CI): 0.043–0.659, *p* = 0.011) was the only significant factor of poor SVR_12_. Fifty-four (5.7%) patients were withdrawn early from the treatment because of hepatic decompensation (n = 18, 1.9%) and other adverse reactions. Multivariate analyses identified old age (odds ratio: 1.062; 95% CI: 1.008–1.119, *p* = 0.024) and Child-Pugh A6 (odds ratio: 4.957; 95% CI: 1.691–14.528, *p* = 0.004) were significantly associated with hepatic decompensation. In conclusion, this large real-world cohort proved PrOD with or without ribavirin to be highly effective in chronic hepatitis C patients with advanced hepatic fibrosis or compensated cirrhosis. However, Child-Pugh A6 should be an exclusion criterion for first-line treatment in these patients.

## Introduction

Hepatitis C virus (HCV) infection is a major cause of chronic liver disease, affecting approximately 150 million people worldwide^1^. Chronic infection with HCV leads to progressive hepatic fibrosis and cirrhosis in around 20% of patients, and 10–20% of cirrhotic patients will develop hepatocellular carcinoma (HCC) within 5 years^1–3^. This implies that HCV eradication is very important in preventing disease progression and associated morbidity and mortality. This is also essential in reducing the future health care burden in society.

Combination of interferon (IFN) and ribavirin (RBV) was the previous standard treatment for chronic hepatitis C. However, lower virologic response rate and lots of side effects with poor adherence limited the application of treatment, and resulted in low sustained virologic response (SVR) rate of 40–50% among patients with HCV genotype 1 (HCV-1) infection^4,5^. The recent advent of direct-acting antiviral agent (DAA) therapy has been widely acknowledged as a revolution in the field of HCV infection. In clinical trials, IFN-free regimens using second generation DAA combinations yield SVR rates above 90% in HCV-1 infected patients^6–8^. Due to high virologic response rates even in difficult-to-treat subgroups such as cirrhosis, non-responders to prior therapy, and transplant recipients, and less side effects with better tolerance, DAA has become the first-line therapy of HCV in the latest guidelines.

Paritaprevir/ritonavir, ombitasvir, and dasabuvir (PrOD)-based regimens for HCV-1 infection have been approved by the Food and Drug Administration (FDA) in the United States (US) since 2016. In one meta-analysis, the SVR rate in PrOD-based regimens with or without RBV can reach up to 94–100% in HCV-1a or HCV-1b patients with and without cirrhosis^7^. However, warning of severe liver injury, hepatic decompensation and even mortality during this treatment were reported and informed by US FDA in 2015^9^. As far, most of the published studies were performed in the US and in Europe; Asian data is still limited^10,11^. In Asia, HCV-1 is the most prevalent form, accounting for 60–70% of HCV infection in Taiwan^12^. Since January 2017, PrOD-based therapies have been reimbursed in Taiwan for HCV-1 patients with advanced hepatic fibrosis (fibrosis 3, F3) or compensated cirrhosis (F4) through a nationwide government-funded program. Thus, we conducted this study to evaluate the safety and efficacy of PrOD-based therapies in a large real-world cohort of patient with advanced hepatic fibrosis or compensated cirrhosis.

## Methods

### Patients and treatments

This was a retrospective cohort study of PrOD-based therapies in patients with HCV-1a or HCV-1b and advanced hepatic fibrosis or compensated cirrhosis from four hospitals in Taiwan (the Keelung, the Linkou, the Chiayi, and the Kaohsiung Chang Gung Memorial Hospital). In Taiwan, patients with HCV and advanced hepatic fibrosis or compensated cirrhosis were treated with DAA via a nationwide government-funded program since 2017. The patients should have positive HCV antibody or detectable HCV RNA in serum for more than 6 months before treatment. Patient who had any evidence of hepatic decompensation or previous exposure to DAA before PrOD-based therapies should be excluded. The provided regimens were 12 weeks of PrOD in HCV-1b with or without cirrhosis, 12 weeks of PrOD plus RBV therapy (PrOD + RBV/12w) in HCV-1a without cirrhosis, and 24 weeks of PrOD plus RBV therapy (PrOD + RBV/24w) in HCV-1a with cirrhosis, respectively. The severity of fibrosis was confirmed by either ultrasonography, fibrosis index based on 4 factors (FIB-4) test, FibroScan (Echosens, Paris, France), or acoustic radiation force impulse (ARFI) (Siemens AG, Erlangen, Germany). Advanced hepatic fibrosis or compensated cirrhosis (Metavir F3-F4) was defined as FIB-4 test ≧3.25, FibroScan ≧9.5 Kpa, or ARFI ≧1.81 m/s.

Patients with BCLC advanced or terminal stage and/or limited life expectancy were excluded. Informed consent was obtained from all patients prior to registration into the program. Demographic data including patient characteristics, treatment information, laboratory studies, and adverse reactions were recorded. For patients with hepatitis B virus (HBV) coinfection, HBV reactivation was defined as either an increase in HBV DNA level of ≥1 log10 IU/mL in patients with baseline detectable HBV DNA level or the HBV DNA level became detectable in patients with undetectable baseline HBV DNA level. Clinically significant hepatitis was defined as an ALT level of ≥3 times upper limit of normal^13^. This study was approved by the Research Ethics Committee of Chang Gung Memorial Hospital and was conducted in accordance with the principles of Declaration of Helsinki and the International Conference on Harmonization for Good Clinical Practice.

### Outcomes

Virologic response (VR) was defined as HCV RNA less than the lower limit of quantification (LLOQ) at week 2, week 4, week 8, and week 12. The primary outcome was SVR_12_ rate, which was defined as the proportion of patients with HCV RNA < LLOQ at post-treatment week 12 in per-protocol population (subjects receiving ≥1 dose of any study medication and HCV RNA data available at post-treatment week 12). The secondary outcome was the early withdrawal rate, which was defined as the percentage of patients who failed to complete the course of PrOD-based therapies because of adverse events, comorbidity, or other reasons. Hepatic decompensation was defined as the presence of clinical events (variceal hemorrhage, and/or ascites, and/or hepatic encephalopathy) or biochemical evidence of worsening liver function (significantly increased total bilirubin >3 mg/dL and/or prolonged prothrombin time ≥3 seconds).

Serum HCV RNA levels were determined by COBAS TaqMan HCV Test (TaqMan HCV; Roche Molecular Systems Inc., Branchburg, N.J., lower limit of detection: 15 IU/ml), or Abbott RealTime HCV assay (ART; Abbott Molecular, Des Plaines, IL; lower limit of detection: 12 IU/ml). Genotyping of HCV was performed by reverse hybridization assay (Inno-LiPA^TM^ HCV II; Innogenetics N.V., Gent, Belgium) using the HCV-Amplicor products, or RealTime Genotyping II RUO assay (Abbott Molecular, Des Plaines, IL). Serum HBV DNA levels were measured using the COBAS AmpliPrep-COBAS TaqMan HBV test (CAP-CTM; Roche Molecular Systems, Inc., Branchburg, NJ, USA), with a detection limit of 15 IU/ml.

### Statistical methods

We used statistical software (SPSS 15.0) for data analysis. Continuous data were expressed as mean ± standard deviation, and categorical data were expressed as number (percentage). In comparing different subgroups, chi-square test or Fisher exact test was used for categorical parameters, and Student’s t-test or Mann–Whitney *U* test was used for continuous parameters where appropriate. Factors related to SVR_12_ and hepatic decompensation were analyzed with univariate and stepwise multivariate logistic regression analyses, and the results were presented as odds ratios (OR) with 95% confidence intervals (CI). Paired *t* test was performed to compare the FIB-4 test between baseline and post-treatment week 12. All statistical tests were 2-tailed, and a *p*-value of less than 0.05 was considered statistically significant.

## Results

### Baseline characteristics

A total of 941 patients were enrolled in this study. The baseline characteristics of the study population are shown in Table 1. Eight hundred eighty-nine (94.5%) patients were infected with HCV-1b, whereas 52 (5.5%) were infected with HCV-1a. The mean age of HCV-1b patients was significantly older than that in HCV-1a patients without cirrhosis (*p* < 0.001) or with cirrhosis (*p* < 0.001). One hundred thirty-one (14%) patients had concomitant HCC, including 79 (8%) without viable tumors and 52 (6%) with viable tumors (active HCC) before PrOD-based therapies. Of the 79 patients without viable tumors, therapy for HCC included 35 local ablation, 31 resection, 7 multiple treatment, 4 transcather arterial chemoembolization and 2 liver transplantation.

**Table 1:** Baseline characteristics.

	Total	PrOD 12 weeks (HCV-1b)	PrOD + RBV 12 weeks (HCV-1a NLC)	PrOD + RBV 24 weeks (HCV-1a LC)
Number	941	889	23	29
Age (yrs)	64.9 ± 9.9	65.4 ± 9.7^ab^	57.1 ± 11.2^a^	58.2 ± 9.3^b^
Male gender, n (%)	442 (47)	412 (46)	11 (48)	19 (66)
Prior IFN, n (%)	543 (58)	512 (58)	13 (57)	18 (62)
HBsAg positive, n (%)	67 (7)	63 (7)	2 (9)	2 (7)
DM, n (%)	177 (19)	170 (19)	4 (17)	3 (10)
HCC, n (%)	131 (14)	128 (14)	1 (4)	2 (6)
No viable tumors, n (%)	79 (8)	77 (8)	1 (4)	1 (3)
Active HCC, n (%)	52 (6)	51 (6)	0 (0)	1 (3)
Fibrosis stage				
advanced fibrosis (F3), n (%)	408 (43)	385 (43)^ab^	23 (100)^a^	0 (0)^b^
compensated cirrhosis A5 (F4), n (%)	468 (50)	443 (50)^ab^	0 (0)^a^	25 (86)^b^
compensated cirrhosis A6 (F4), n (%)	65 (7)	61 (7)	0 (0)	4 (14)
Albumin (g/dl)	4.2 ± 0.4	4.2 ± 0.4^a^	4.4 ± 0.3^a^	4.2 ± 0.4
Total bilirubin (mg/dl)	0.9 ± 0.4	0.9 ± 0.4	0.9 ± 0.4	1.1 ± 0.5
AST (U/L)	85 ± 59	85 ± 55^a^	118 ± 148^a^	74 ± 49
ALT (U/L)	87 ± 67	87 ± 68	101 ± 72	67 ± 39
Platelet (10^3^/μL)	137 ± 57	136 ± 56^a^	185 ± 67^a^	135 ± 63
FIB-4	5.1 ± 4.3	5.2 ± 4.3^a^	3. ± 1.6^a^	4.3 ± 3.0
HCV RNA > 8*10^5^ IU/mL, n (%)	623 (66)	589 (66)	15 (65)	19 (66)

Data are expressed as mean ± standard deviation or number (percentage).

Abbreviation: PrOD, paritaprevir/ritonavir, ombitasvir, and dasabuvir; RBV, ribavirin; NLC, non-cirrhosis; LC, cirrhosis; IFN, interferon; HBsAg, hepatitis B surface antigen; DM, diabetes mellitus; HCC, hepatocellular carcinoma; A5, Child-Pugh A5; A6, Child-Pugh A6; AST, aspartate aminotransferase; ALT, alanine aminotransferase; FIB-4, fibrosis index based on 4 factors; HCV, hepatitis C virus.

^a^Significant differences between HCV-1b and HCV-1a NLC.

^b^Significant differences between HCV-1b and HCV-1a LC.

### Efficacy outcome

**Figure 1:**
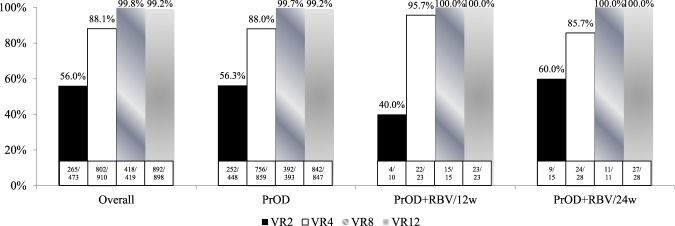
On-treatment virological response during PrOD-based therapies. PrOD: HCV-1b with and without cirrhosis; PrOD + RBV/12w: HCV-1a without cirrhosis; PrOD + RBV/24w: HCV-1a with cirrhosis.

**Figure 2:**
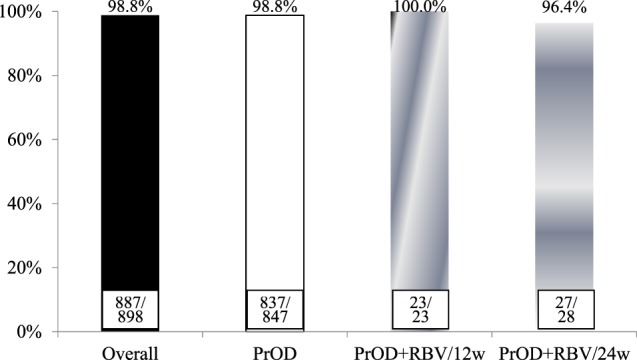
Overall SVR_12_ rate. PrOD: HCV-1b with and without cirrhosis; PrOD + RBV/12w: HCV-1a without cirrhosis; PrOD + RBV/24w: HCV-1a with cirrhosis.

A total of 887 patients completed the course of PrOD-based therapies. Of the patients with available data, the overall VR2 rate, VR4 rate, VR8 rate, and VR12 rate were 56%, 88.1%, 99.8%, and 99.2%, respectively (Fig. 1). There were no significant differences among patients with HCV-1b, HCV-1a non-cirrhosis and HCV-1a cirrhosis. Based on the per-protocol analysis, the overall SVR_12_ rate were 98.8%, 100%, and 96.4% in patients with HCV-1b, HCV-1a non-cirrhosis and HCV-1a cirrhosis, respectively (Fig. 2). As shown in Table 2, univariate analysis revealed that Child-Pugh A6 (*p* = 0.016) and lower serum albumin level (*p* = 0.003) were associated with poor SVR_12_ rate. Stepwise multivariate analysis demonstrated that Child-Pugh A6 (OR: 0.168, 95% CI: 0.043–0.659; *p* = 0.011) was independently associated with poor SVR_12_.

**Table 2:** Univariate and multivariate analyses of factors associated with SVR_12_ after PrOD-based therapies.

	Comparison	Univariate analyses		Stepwise multivariate analyses	
		OR (95% CI)	p-value	OR (95% CI)	p-value
Age	per 1 year increase	0.994 (0.936–1.056)	0.857	—	—
Gender	Male vs. female	0.340 (0.090–1.291)	0.113	—	—
Prior IFN	Yes vs. no	0.138 (0.018–1.081)	0.059	0.130 (0.016–1.025)	0.053
DM	Yes vs. no	0.637 (0.167–2.426)	0.508	—	—
Genotype	1a vs. 1b	0.597 (0.075–4.759)	0.627	—	—
Child-Pugh	A6 vs. non-A6	0.176 (0.045–0.683)	0.016	0.168 (0.043–0.659)	0.011
Albumin (g/dl)	per 1 g/dl increase	4.645 (1.158–18.63)	0.030	—	—
Total bilirubin (mg/dl)	per 1 mg/dl increase	0.691 (0.149–3.198)	0.636	—	—
AST (U/L)	per 1 U/L increase	1.014 (0.996–1.033)	0.126	—	—
ALT (U/L)	per 1 U/L increase	1.016 (0.997–1.035)	0.099	—	—
Platelet (10^3^/μL)	per 10^3^/μL increase	1.000 (0.990–1.011)	0.957	—	—
FIB-4	per 1 increase	0.985 (0.870–1.115)	0.808	—	—

Abbreviation: SVR, sustained virological response; PrOD, paritaprevir/ritonavir, ombitasvir, and dasabuvir; OR, odds ratio; CI, confidence interval; IFN, interferon; DM, diabetes mellitus; AST, aspartate aminotransferase; ALT, alanine aminotransferase; FIB-4, fibrosis index based on 4 factors.

After PrOD-based therapies, FIB-4 test significantly decreased from 5.1 ± 4.3 at baseline to 3.4 ± 2.4 at post-treatment week 12 (*p* < 0.001). These changes were all significant among three subgroup patients of advanced hepatic fibrosis (4.0 ± 3.4 to 2.8 ± 1.5, *p* < 0.001), compensated A5 cirrhosis (5.3 ± 3.7 to 3.6 ± 2.3, *p* < 0.001) and compensated A6 cirrhosis (9.6 ± 7.7 to 5.9 ± 4.7, *p* < 0.001), respectively.

### Safety outcome

Of the study population, 54 (5.7%) patients prematurely discontinued the treatment due to adverse events or comorbidity of the underlying disease. Three patients died from severe infection (1 spontaneous bacterial peritonitis, 1 infected prosthetic dialysis arteriovenous grafts, and 1 pneumonia) during therapy. Two patients died from ruptured HCC and acute myocardial infarction during follow-up, respectively. Other causes of early withdrawal included hepatic decompensation (n = 18), nausea and general malaise (n = 12), poor adherence (n = 5), drug-drug interaction (n = 3), ALT ≥10 times upper normal limit (n = 2), and other reasons in the remaining 9 patients. The predictors of hepatic decompensation were analyzed in Table 3. By univariate analyses, old age (*p* = 0.015), concomitant HCC (*p* = 0.023), Child-Pugh A6 (*p* = 0.002), lower serum albumin level (*p* < 0.001), higher total bilirubin level (*p* = 0.004) and lower platelet count (*p* = 0.015) were associated with the development of hepatic decompensation during PrOD-based therapies. Multivariate analyses showed that old age (OR: 1.062; 95% CI: 1.008–1.119, *p* = 0.024) and Child-Pugh A6 (OR: 4.957; 95% CI: 1.691–14.528, *p* = 0.004) were independent variables.

**Table 3:** Univariate and multivariate analyses of factors predicting hepatic decompensation during PrOD-based therapies.

	Comparison	Univariate analyses		Stepwise multivariate analyses	
		OR (95% CI)	p-value	OR (95% CI)	p-value
Age	per 1 year increase	1.066 (1.012–1.122)	0.015	1.062 (1.008–1.119)	0.024
Gender	Male vs. female	0.558 (0.208–1.501)	0.248	—	—
Prior IFN	Yes vs. no	1.476 (0.549–3.968)	0.440	—	—
DM	Yes vs. no	1.594 (0.561–4.532)	0.382	—	—
HCC	Yes vs. no	3.188 (1.175–8.648)	0.023	—	—
Child-Pugh	A6 vs. non-A6	5.532 (1.909–16.03)	0.002	4.957 (1.691–14.528)	0.004
Albumin (g/dl)	per 1 g/dl increase	0.124 (0.044–0.347)	<0.001	—	—
Total bilirubin (mg/dl)	per 1 mg/dl increase	3.916 (1.536–9.987)	0.004	—	—
AST (U/L)	per 1 U/L increase	0.996 (0.986–1.006)	0.431	—	—
ALT (U/L)	per 1 U/L increase	0.993 (0.976–0.997)	0.214	—	—
Platelet (10^3^/μL)	per 10^3^/μL increase	0.987 (0.990–1.011)	0.015	—	—
FIB-4	per 1 increase	1.061 (0.997–1.130)	0.061	—	—

Abbreviation: PrOD, paritaprevir/ritonavir, ombitasvir, and dasabuvir; OR, odds ratio; CI, confidence interval; IFN, interferon; DM, diabetes mellitus; AST, aspartate aminotransferase; ALT, alanine aminotransferase; FIB-4, fibrosis index based on 4 factors.

Table 4 summarizes the characteristics of 18 patients with on-treatment hepatic decompensation during PrOD-based therapies. Among these patients, 6 (33%) were male, 12 (67%) were treatment-experienced, 6 (33%) had HCC, and 5 (28%) had Child-Pugh A6 cirrhosis. None were related to HBV reactivation. The mean of onset of hepatic decompensation was 13.1 ± 7.9 (6~28) days, and the mean of timing for recovery was 34.4 ± 42.9 (6~170) days. Three patients achieved an SVR_12_ after 32.7 ± 8.1 days of therapy.

**Table 4:** Summary of patients characteristics with on-treatment hepatic decompensation during PrOD-based therapies.

	Decompensation (n = 18)
Age (years)	70.6 ± 9.3 (54~89)
Male gender, n (%)	6 (33)
Prior IFN, n (%)	12 (67)
HBsAg positive, n (%)	0 (0)
DM, n (%)	5 (28)
HCC, n (%) Active HCC, n (%)	6 (33) 4 (22)
Genotype 1a, n (%)	0 (0)
Fibrosis stage	
advanced fibrosis (F3), n (%)	6 (33)
compensated cirrhosis A5 (F4), n (%)	7 (39)
compensated cirrhosis A6 (F4), n (%)	5 (28)
Onset of hepatic decompensation (days)	13.1 ± 7.9 (6~28)
Timing of stopping PrOD therapy (days)	20.8 ± 17.8 (6~71)
Timing of recovery	34.4 ± 42.9 (6~170)
SVR_12_, n (%)*	3 (17)

Data are expressed as mean ± standard deviation or number (percentage).

Abbreviation: PrOD, Paritaprevir/ritonavir, ombitasvir, and dasabuvir; IFN, interferon; DM, diabetes mellitus; HCC, hepatocellular carcinoma.

*Data not available in 15 patients.

**Figure 3:**
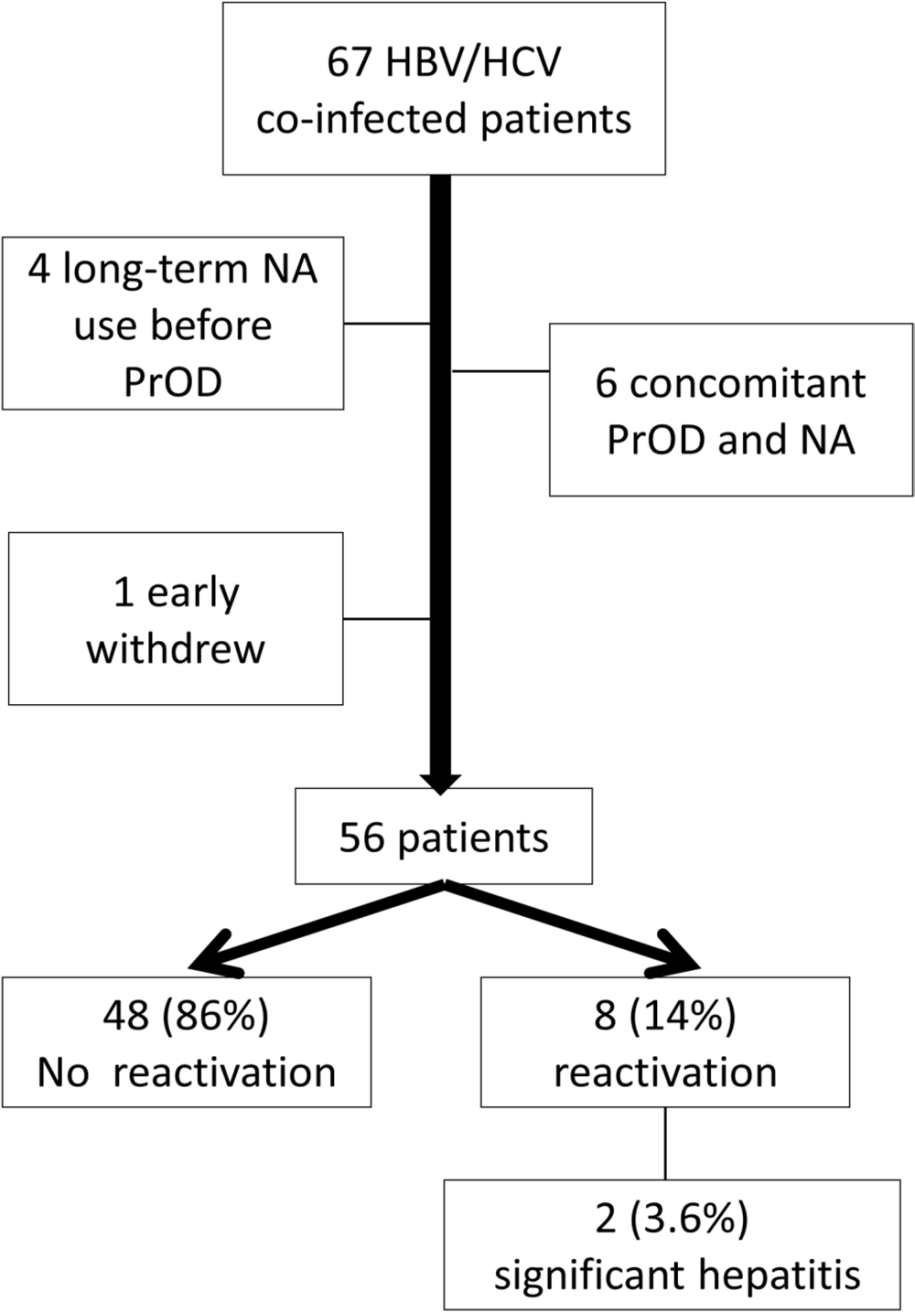
Patients co-infected with HBV and HCV received PrOD-based therapies.

Among 67 patients with HBV coinfection, 4 had long-term use of nucleos(t)ide analogue (NA) before PrOD-based therapies. The rest of the 63 patients included 30 (48%) with undetectable HBV DNA level and 33 (52%) patients with detectable HBV DNA level at baseline. Six patients received concomitant PrOD and NA treatment (3 with HBV DNA level of ≥2000 IU/ml and 3 with HBV DNA level <2000 IU/ml). After excluding these patients with prior or concomitant NA treatment and 1 patient with early withdrawal, 8 (14%) patients met the virologic criteria for HBV reactivation (Fig. 3). Of them, two patients (3.6%) with HBV reactivation and clinically significant hepatitis received NA immediately and did not develop hepatic decompensation.

## Discussion

This is one of the largest real-world cohort studies enrolling HCV-1 patients with advanced hepatic fibrosis or compensated cirrhosis receiving PrOD-based therapies. Such studies are of great clinical importance, since safety and effectiveness are often lower than in clinical trials which usually include only highly selected patients^14^. In particular, safety concerns have been raised in cirrhotic patients treated with PrOD-based therapies. In our study, the overall SVR_12_ rate was 98.8% in HCV-1b, 100% in HCV-1a without cirrhosis, and 96.4% in HCV-1a with cirrhosis. These results were comparable to those in previous clinical trials^15–18^.

Previous real-world studies have demonstrated that PrOD-based therapies achieved SVR_12_ rate of 95% to 100% in HCV-1b with or without cirrhosis, 95% to 100% in HCV-1a without cirrhosis, and 92.6% to 93% in HCV-1a with cirrhosis^19–24^. However, African-American patients were reported to have a lower SVR_12_ rate compared to Caucasian patients (89.8% vs. 92.8%; *p* = 0.003) from an US cohort^23^. In contrast, our data concurring with other studies conducted in East Asia^10,11^ confirmed the effectiveness of PrOD without RBV in Asian patients with HCV-1b infection, and PrOD with RBV in HCV-1a infection.

Our study consisted of a large database of homogenous patients, thus we could identify the possible predictive factors of SVR_12_ to PrOD-based therapies despite the high SVR_12_ rate. In our study, multivariate analysis showed the presence of Child-Pugh A6 had a negative impact on SVR_12_ independently. Although reasons for the lower SVR rate in Child-Pugh A6 patients need to be further clarified, several mechanisms may be proposed. Advanced cirrhotic changes and shunting could result in the inadequacy of drug delivery^25–27^, and uptake and metabolism might be affected by shunting and poor liver function^28,29^. In addition, viral clearance might be impaired as a result of immune defects caused by more advanced cirrhosis^30,31^. Furthermore, Child-Pugh A6 cirrhosis significantly worsens tolerability of treatment leading to high rates of treatment discontinuation.

Previous studies have reported that the rate of adverse reaction, the rate of severe adverse reaction and the early withdrawal rate during the PrOD-based therapy was 42% to 91%, 1.7% to 10.3% and 0% to 6.3%, respectively^19–24^. In our cohort, 54 patients (5.7%) prematurely discontinued the treatment due to serious adverse events or comorbidity of the underlying disease. This result was not inferior to those in previous studies even though all of our patients had advanced hepatic fibrosis or compensated cirrhosis. Importantly, hepatic decompensation is the most serious complication during the PrOD-based therapies. Overall, the incidence of hepatic decompensation in our cohort was 2% (n = 18), but there was no mortality associated with PrOD-based therapies. This was relatively different from one Romania cohort showing that the mortality rate had reached as high as 35% in the patients presenting with hepatic decompensation^24^. The causes of hepatic decompensation were related to the baseline characteristics of the patient, including advanced liver disease or with a history of hepatic decompensation, and idiosyncratic drug-related liver injury. In our study, multivariate analysis showed that old age and the presence of Child-Pugh A6 predicted hepatic decompensation independently. When the patients developed liver decompensation during the PrOD-based therapies, although some patients could still achieve viral eradication despite the shorter treatment duration, the frequency and severity of adverse events would increase significantly, and even life-threatening^32^. Thus, immediate discontinuation of PrOD is suggested when hepatic decompensation develops, and retreatment by another DAA is strongly recommended if needed.

The potential drug-drug interaction (DDI) is another disadvantage of PrOD, which inhibits multiple isoenzymes of the cytochrome P-450 family (CYP2C19 and CYP3A4) and may therefore affect the metabolism of many drugs^7^. These interactions may lead to unsafe levels of concomitant medications or loss of PrOD efficacy. Liu *et al*. recently reported that the prevalence of contraindications with PrOD was 13.3% in Taiwanese patients^33^. Importantly, elderly patients were more likely to have potential DDI due to increasing use of concomitant drugs^33^. It is highly recommended to increase awareness of potential DDI during PrOD-based therapies, especially in patients with impaired liver function and old age.

Of note, the US FDA has mandated the addition of a boxed warning to remind practitioners of the potential HBV reactivation and its related complications during IFN-free DAAs for HCV^34^. Our study showed that two patients (3.6%) HBV-coinfected patients with HBV reactivation presented clinically significant hepatitis but did not develop hepatic decompensation. This result was in accordance with a recent prospective study^35^, showing that most patients with evidence of HBV reactivation were asymptomatic and could be managed with watchful surveillance or oral antiviral agents for HBV.

Our study enrolled a large number of patients, giving us a chance to study the safety and efficiency of PrOD-based therapies in the elderly patients with advanced liver disease. However, there were still some limitations. First, due to its retrospective design, the mild to moderate adverse events might be underreported. Second, our study did not analyze the resistance-associated substitution (RAS) for all patients at the start of and when treatment failed. However, based on the evidence that HCV-1 patients treated with PrOD achieved similarly high SVR rates, regardless of the presence or absence of baseline RASs, current guidelines advised against routine check of baseline RASs in these patients^36^. Third, the number of patients in the HCV-1a subgroup was relatively low in this area. While our study appeared to be the largest cohort enrolling HCV-1a patients in Asia, since the prevalence of HCV-1a was low in this area.

In conclusion, our large real-world cohort suggests that PrOD with or without RBV is highly effective in Asian patients with advanced hepatic fibrosis or compensated cirrhosis, achieving an SVR_12_ rate of 98.8%. Child-Pugh A6 not only correlated with poor SVR_12_ rate but also predicted hepatic decompensation during PrOD-based therapies. Therefore, this should be an exclusion criterion for first-line treatment in these patients.
